# *N*,*N*′-Diphenyl­thio­urea acetone monosolvate

**DOI:** 10.1107/S1600536810050300

**Published:** 2010-12-08

**Authors:** Andrzej Okuniewski, Jaroslaw Chojnacki, Barbara Becker

**Affiliations:** a Department of Inorganic Chemistry, Gdansk University of Technology, 11/12 Narutowicza Str., 80-233 Gdańsk, Poland

## Abstract

In the title compound, C_13_H_12_N_2_S·C_3_H_6_O, the phenyl rings of the thio­urea mol­ecule are in *syn* and *anti* positions in relation to the C=S bond. Two mol­ecules are connected by N—H⋯S=C hydrogen bonds into a centrosymmetric dimer. An additional N—H⋯O=C hydrogen bond to the acetone solvent mol­ecule and some weak C—H⋯π inter­actions reinforce the crystal structure.

## Related literature

For the unsolvated *N*,*N′*-diphenyl­thio­urea stereoisomers, see: Ramnathan *et al.* (1995 ▶); Peseke *et al.* (1999 ▶). For the *syn*-*syn*-*N*,*N′*-diphenyl­thio­urea–dicyclo­hexyl-18-crown-6 co-crystal, see: Fonari *et al.* (2005 ▶). For related structures, see: Bowmaker *et al.* (2009 ▶); Okuniewski *et al.* (2010 ▶); Shen & Xu (2004 ▶).
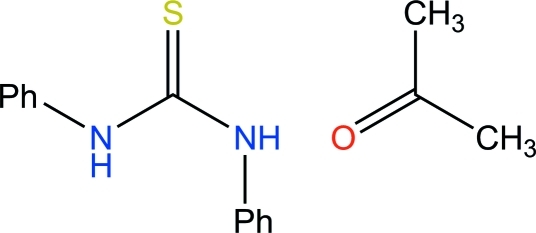


## Experimental

### 

#### Crystal data

C_13_H_12_N_2_S·C_3_H_6_O
*M* *_r_* = 286.38
Orthorhombic, 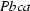

*a* = 17.1797 (6) Å
*b* = 10.0736 (4) Å
*c* = 17.4700 (7) Å
*V* = 3023.4 (2) Å^3^
*Z* = 8
Mo *K*α radiation
μ = 0.21 mm^−1^
*T* = 150 K
0.46 × 0.41 × 0.27 mm

#### Data collection

Oxford Diffraction Xcalibur Sapphire2 diffractometer
Absorption correction: analytical (*CrysAlis PRO*; Oxford Diffraction, 2009 ▶) *T*_min_ = 0.777, *T*_max_ = 0.819
7549 measured reflections
3245 independent reflections
2278 reflections with *I* > 2σ(*I*)
*R*_int_ = 0.025

#### Refinement

*R*[*F* ^2^ > 2σ(*F* ^2^)] = 0.039
*wR*(*F* ^2^) = 0.092
*S* = 0.94
3245 reflections
191 parameters
2 restraints
H atoms treated by a mixture of independent and constrained refinement
Δρ_max_ = 0.32 e Å^−3^
Δρ_min_ = −0.21 e Å^−3^

### 

Data collection: *CrysAlis PRO* (Oxford Diffraction, 2009 ▶); cell refinement: *CrysAlis PRO*; data reduction: *CrysAlis PRO*; program(s) used to solve structure: *SHELXS97* (Sheldrick, 2008 ▶); program(s) used to refine structure: *SHELXL97* (Sheldrick, 2008 ▶); molecular graphics: *OLEX2* (Dolomanov *et al.*, 2009 ▶); software used to prepare material for publication: *WinGX* (Farrugia, 1999 ▶) and *PLATON* (Spek, 2009 ▶).

## Supplementary Material

Crystal structure: contains datablocks global, I. DOI: 10.1107/S1600536810050300/ng5077sup1.cif

Structure factors: contains datablocks I. DOI: 10.1107/S1600536810050300/ng5077Isup2.hkl

Additional supplementary materials:  crystallographic information; 3D view; checkCIF report

## Figures and Tables

**Table 1:** Hydrogen-bond geometry (Å, °) *Cg*1 and *Cg*2 are the centroids of the C11–C16 and C21–C26 rings, respectively.

*D*—H⋯*A*	*D*—H	H⋯*A*	*D*⋯*A*	*D*—H⋯*A*
N1—H1⋯S1^i^	0.87 (1)	2.48 (1)	3.3240 (13)	165 (1)
N2—H2⋯O1	0.87 (1)	2.09 (1)	2.8993 (18)	154 (2)
C2—H2*A*⋯*Cg*1	0.98	3.02	3.931 (2)	155
C2—H2*C*⋯*Cg*2^ii^	0.98	2.80	3.607 (2)	140

Symmetry codes: (i) 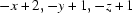; (ii) 
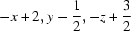
.

**Fig. 1.**
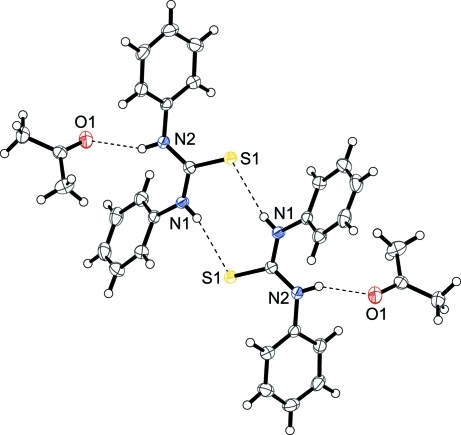
Structure of [SC(NHPh)2.OC(CH3)2]2 dimer.

## supplementary crystallographic information

### Comment

*N*,*N'*-Diphenylthiourea (thiocarbanilide), S═C(NHPh)_2_, is commonly used as rubber vulcanization accelerator and as a stabilizer for PVC and PVDC. X-ray structures of its two possible stereoisomers have been already determined. The first is *syn*-*syn* isomer (Ramnathan *et al.*, 1995), where only weak R_2_^1^(6) bifurcative N—H···S hydrogen bonds are present. The second – *syn*-*anti* isomer (Peseke *et al.*, 1999) – is more stable because of dimer formation. Two N—H···S hydrogen bonds form R_2_^2^(8) centrosymmetric structural motif. There are also some π···π and N—H···π interactions.

CSD 5.32 contains data on 15 structures of *N*,*N'*-diphenylthiourea and its complexes. *Syn*-*anti* isomer is more common (12 cases). *Syn*-*syn* isomer is present in the single-component crystal (Ramnathan *et al.*, 1995), as a cocrystal with dicyclohexyl-18-crown-6 (Fonari *et al.*, 2005) and as a copper(I) complex (Bowmaker *et al.*, 2009).

When *N*,*N'*-diphenylthiourea cocrystalizes with acetone in *Pbca* space group the centrosymmetric dimer is also formed as it is common among compounds containing S═C*R*^1^—N*R*^2^—H group. There is at least 109 such structures deposited in CSD. This motif is particularly common among *N*-acyl-*N'*-arylureas and thioureas (Okuniewski *et al.*, 2010). When monosubstituted *N*-phenylthiourea is considered, chains of molecules can be found (Shen & Xu, 2004). In the title compound an additional N—H···O═C hydrogen bond to acetone is formed stabilizing the structure. Crystals are well formed and grow up to several milimeters in just one day.

There is no π···π stacking in this structure, but some weak C—H···π interactions can be found (see Tab. 1).

Melting point, 154°C, is the same as that for pure *N*,*N*'-diphenylthiourea. This is because crystals very quickly loose acetone molecules before melting (even at room temperature) and became colourless powder of pure thiourea derivative.

### Experimental

1.82 g (8 mmol) of commercially available *N*,*N'*-diphenylthiourea was added to 25 ml of acetone and gently heated while stirring. After 5 min nearly full dissolution was observed. The mixture was allowed to cool and then was filtered. The filtrate was left for crystalization at room temperature. After one day well formed, colourless shiny crystals were collected. Yield – 1.86 g (81%).

### Refinement

Hydrogen atoms were placed at calculated positions (*d*_CH_ = 0.95–0.98 Å) and were treated as riding on their parent atoms, with *U*(H) set to 1.2–1.5 times *U*_eq_(C). The N—H distances were restrained to 0.88 (1) Å.

### Crystal data

**Table:**

C_13_H_12_N_2_S·C_3_H_6_O	*D*_x_ = 1.258 Mg m^−^^3^
*M**_r_* = 286.38	Melting point: 154(1) K
Orthorhombic, *P**b**c**a*	Mo *K*α radiation, λ = 0.71073 Å
Hall symbol: -P 2ac 2ab	Cell parameters from 3367 reflections
*a* = 17.1797 (6) Å	θ = 2.3–28.7°
*b* = 10.0736 (4) Å	µ = 0.21 mm^−^^1^
*c* = 17.4700 (7) Å	*T* = 150 K
*V* = 3023.4 (2) Å^3^	Prism, clear colourless
*Z* = 8	0.46 × 0.41 × 0.27 mm
*F*(000) = 1216	

### Data collection

**Table:**

Oxford Diffraction Xcalibur Sapphire2 diffractometer	3245 independent reflections
Radiation source: fine-focus sealed tube	2278 reflections with *I* > 2σ(*I*)
graphite	*R*_int_ = 0.025
Detector resolution: 8.1883 pixels mm^-1^	θ_max_ = 27°, θ_min_ = 2.6°
ω scans	*h* = −9→21
Absorption correction: analytical (*CrysAlis PRO*; Oxford Diffraction, 2009)	*k* = −7→12
*T*_min_ = 0.777, *T*_max_ = 0.819	*l* = −22→17
7549 measured reflections	

### Refinement

**Table:**

Refinement on *F*^2^	Primary atom site location: structure-invariant direct methods
Least-squares matrix: full	Secondary atom site location: difference Fourier map
*R*[*F*^2^ > 2σ(*F*^2^)] = 0.039	Hydrogen site location: inferred from neighbouring sites
*wR*(*F*^2^) = 0.092	H atoms treated by a mixture of independent and constrained refinement
*S* = 0.94	*w* = 1/[σ^2^(*F*_o_^2^) + (0.054*P*)^2^] where *P* = (*F*_o_^2^ + 2*F*_c_^2^)/3
3245 reflections	(Δ/σ)_max_ = 0.001
191 parameters	Δρ_max_ = 0.32 e Å^−^^3^
2 restraints	Δρ_min_ = −0.21 e Å^−^^3^

### Special details

**Table:**

Geometry. All s.u.'s (except the s.u. in the dihedral angle between two l.s. planes) are estimated using the full covariance matrix. The cell s.u.'s are taken into account individually in the estimation of s.u.'s in distances, angles and torsion angles; correlations between s.u.'s in cell parameters are only used when they are defined by crystal symmetry. An approximate (isotropic) treatment of cell s.u.'s is used for estimating s.u.'s involving l.s. planes.The phenyl rings centroids: *Cg*1 is the centroid of ring {C11,..,C16}: 0.22445 (4), 0.73429 (8), 0.46519 (4); *Cg*2 is the centroid of ring {C21,..,C26}: 0.06414 (4), 0.39968 (8), 0.17172 (4). Distance calculations were done using *PLATON* (Spek, 2009).
Refinement. Refinement of *F*^2^ against ALL reflections. The weighted *R*-factor *wR* and goodness of fit *S* are based on *F*^2^, conventional *R*-factors *R* are based on *F*, with *F* set to zero for negative *F*^2^. The threshold expression of *F*^2^ > 2σ(*F*^2^) is used only for calculating *R*-factors(gt) *etc*. and is not relevant to the choice of reflections for refinement. *R*-factors based on *F*^2^ are statistically about twice as large as those based on *F*, and *R*- factors based on ALL data will be even larger.

### Fractional atomic coordinates and isotropic or equivalent isotropic displacement parameters (Å^2^)

**Table:**

	*x*	*y*	*z*	*U*_iso_*/*U*_eq_	
S1	1.00623 (2)	0.60332 (4)	0.60875 (2)	0.02588 (12)	
N1	0.90820 (7)	0.42124 (13)	0.56131 (7)	0.0227 (3)	
H1	0.9367 (8)	0.4256 (17)	0.5206 (7)	0.033 (5)*	
N2	0.89857 (8)	0.47225 (14)	0.68979 (7)	0.0245 (3)	
H2	0.8677 (9)	0.4042 (13)	0.6956 (10)	0.040 (5)*	
C1	0.93269 (8)	0.49352 (16)	0.62170 (8)	0.0219 (3)	
C11	0.84080 (9)	0.34076 (16)	0.55234 (8)	0.0238 (3)	
C12	0.84703 (10)	0.23117 (18)	0.50489 (8)	0.0292 (4)	
H12	0.8963	0.207	0.4846	0.035*	
C13	0.78192 (11)	0.1567 (2)	0.48687 (10)	0.0389 (5)	
H13	0.7864	0.0819	0.454	0.047*	
C14	0.71028 (11)	0.1916 (2)	0.51682 (10)	0.0406 (5)	
H14	0.6653	0.1414	0.5042	0.049*	
C15	0.70426 (9)	0.2990 (2)	0.56486 (10)	0.0358 (4)	
H15	0.655	0.3215	0.586	0.043*	
C16	0.76902 (9)	0.37509 (18)	0.58304 (9)	0.0291 (4)	
H16	0.7643	0.4496	0.616	0.035*	
C21	0.91899 (8)	0.53867 (17)	0.75969 (8)	0.0238 (4)	
C22	0.91081 (9)	0.67464 (17)	0.76642 (9)	0.0285 (4)	
H22	0.8934	0.7256	0.724	0.034*	
C23	0.92812 (10)	0.73653 (19)	0.83536 (10)	0.0350 (4)	
H23	0.9229	0.8301	0.84	0.042*	
C24	0.95281 (10)	0.6625 (2)	0.89701 (9)	0.0376 (5)	
H24	0.9645	0.7048	0.9442	0.045*	
C25	0.96061 (11)	0.5259 (2)	0.88998 (9)	0.0371 (5)	
H25	0.9775	0.4749	0.9326	0.045*	
C26	0.94387 (9)	0.46364 (19)	0.82118 (8)	0.0305 (4)	
H26	0.9495	0.3702	0.8164	0.037*	
O1	0.79841 (7)	0.27541 (12)	0.76020 (7)	0.0398 (3)	
C1A	0.80931 (9)	0.15731 (18)	0.76927 (9)	0.0273 (4)	
C2	0.85919 (11)	0.0803 (2)	0.71495 (9)	0.0404 (5)	
H2A	0.8556	0.1196	0.6638	0.061*	
H2B	0.8413	−0.012	0.7131	0.061*	
H2C	0.9134	0.0829	0.7324	0.061*	
C3	0.77585 (10)	0.08360 (18)	0.83558 (9)	0.0328 (4)	
H3A	0.7408	0.1422	0.8642	0.049*	
H3B	0.818	0.0538	0.8692	0.049*	
H3C	0.7467	0.0064	0.8169	0.049*	

### Atomic displacement parameters (Å^2^)

**Table:**

	*U*^11^	*U*^22^	*U*^33^	*U*^12^	*U*^13^	*U*^23^
S1	0.0267 (2)	0.0296 (2)	0.0214 (2)	−0.00829 (19)	0.00224 (15)	−0.00198 (18)
N1	0.0218 (6)	0.0268 (8)	0.0194 (6)	−0.0039 (6)	0.0019 (5)	−0.0024 (6)
N2	0.0279 (7)	0.0255 (8)	0.0200 (6)	−0.0065 (6)	0.0023 (5)	0.0005 (6)
C1	0.0222 (7)	0.0213 (8)	0.0222 (7)	0.0028 (7)	−0.0004 (6)	0.0007 (7)
C11	0.0266 (8)	0.0249 (9)	0.0199 (7)	−0.0051 (7)	−0.0018 (6)	0.0039 (7)
C12	0.0336 (8)	0.0284 (10)	0.0257 (8)	−0.0036 (8)	−0.0011 (7)	−0.0001 (8)
C13	0.0503 (11)	0.0336 (11)	0.0329 (9)	−0.0142 (9)	−0.0068 (8)	−0.0030 (9)
C14	0.0400 (10)	0.0423 (12)	0.0394 (10)	−0.0205 (10)	−0.0102 (8)	0.0107 (10)
C15	0.0253 (8)	0.0433 (12)	0.0388 (10)	−0.0074 (8)	0.0000 (7)	0.0113 (9)
C16	0.0268 (8)	0.0315 (10)	0.0289 (8)	−0.0016 (8)	0.0002 (6)	0.0014 (8)
C21	0.0215 (7)	0.0304 (9)	0.0196 (7)	−0.0055 (7)	0.0036 (6)	−0.0007 (7)
C22	0.0311 (9)	0.0295 (10)	0.0250 (8)	0.0003 (8)	0.0013 (6)	−0.0004 (8)
C23	0.0344 (9)	0.0341 (10)	0.0364 (9)	−0.0039 (8)	0.0050 (7)	−0.0106 (8)
C24	0.0355 (10)	0.0542 (13)	0.0230 (8)	−0.0118 (10)	0.0016 (7)	−0.0090 (9)
C25	0.0400 (10)	0.0494 (12)	0.0219 (8)	−0.0137 (10)	−0.0030 (7)	0.0092 (9)
C26	0.0330 (9)	0.0323 (10)	0.0264 (8)	−0.0075 (8)	−0.0011 (7)	0.0044 (8)
O1	0.0481 (8)	0.0297 (7)	0.0416 (7)	−0.0060 (6)	0.0094 (6)	0.0028 (6)
C1A	0.0249 (8)	0.0307 (10)	0.0263 (8)	−0.0053 (8)	−0.0050 (6)	−0.0019 (8)
C2	0.0403 (10)	0.0517 (13)	0.0292 (9)	0.0064 (10)	−0.0015 (7)	−0.0017 (9)
C3	0.0304 (9)	0.0352 (11)	0.0329 (9)	−0.0052 (8)	−0.0004 (7)	0.0065 (8)

### Geometric parameters (Å, °)

**Table:**

S1—C1	1.6943 (16)	C21—C22	1.382 (2)
N1—C1	1.3492 (18)	C22—C23	1.388 (2)
N1—C11	1.4221 (19)	C22—H22	0.95
N1—H1	0.865 (9)	C23—C24	1.377 (3)
N2—C1	1.3433 (18)	C23—H23	0.95
N2—C21	1.4359 (19)	C24—C25	1.387 (3)
N2—H2	0.873 (9)	C24—H24	0.95
C11—C12	1.385 (2)	C25—C26	1.386 (2)
C11—C16	1.389 (2)	C25—H25	0.95
C12—C13	1.383 (2)	C26—H26	0.95
C12—H12	0.95	O1—C1A	1.215 (2)
C13—C14	1.383 (3)	C1A—C3	1.491 (2)
C13—H13	0.95	C1A—C2	1.495 (2)
C14—C15	1.373 (3)	C2—H2A	0.98
C14—H14	0.95	C2—H2B	0.98
C15—C16	1.388 (2)	C2—H2C	0.98
C15—H15	0.95	C3—H3A	0.98
C16—H16	0.95	C3—H3B	0.98
C21—C26	1.381 (2)	C3—H3C	0.98
			
C1—N1—C11	130.37 (13)	C21—C22—C23	119.81 (16)
C1—N1—H1	116.0 (11)	C21—C22—H22	120.1
C11—N1—H1	113.6 (11)	C23—C22—H22	120.1
C1—N2—C21	124.89 (14)	C24—C23—C22	120.07 (18)
C1—N2—H2	119.5 (12)	C24—C23—H23	120
C21—N2—H2	114.6 (11)	C22—C23—H23	120
N2—C1—N1	118.05 (14)	C23—C24—C25	119.85 (16)
N2—C1—S1	123.22 (11)	C23—C24—H24	120.1
N1—C1—S1	118.71 (11)	C25—C24—H24	120.1
C12—C11—C16	119.89 (15)	C26—C25—C24	120.37 (17)
C12—C11—N1	117.23 (14)	C26—C25—H25	119.8
C16—C11—N1	122.59 (15)	C24—C25—H25	119.8
C13—C12—C11	120.40 (16)	C21—C26—C25	119.40 (17)
C13—C12—H12	119.8	C21—C26—H26	120.3
C11—C12—H12	119.8	C25—C26—H26	120.3
C14—C13—C12	119.74 (18)	O1—C1A—C3	121.98 (16)
C14—C13—H13	120.1	O1—C1A—C2	120.89 (16)
C12—C13—H13	120.1	C3—C1A—C2	117.11 (16)
C15—C14—C13	119.91 (17)	C1A—C2—H2A	109.5
C15—C14—H14	120	C1A—C2—H2B	109.5
C13—C14—H14	120	H2A—C2—H2B	109.5
C14—C15—C16	120.98 (16)	C1A—C2—H2C	109.5
C14—C15—H15	119.5	H2A—C2—H2C	109.5
C16—C15—H15	119.5	H2B—C2—H2C	109.5
C15—C16—C11	119.07 (17)	C1A—C3—H3A	109.5
C15—C16—H16	120.5	C1A—C3—H3B	109.5
C11—C16—H16	120.5	H3A—C3—H3B	109.5
C26—C21—C22	120.50 (15)	C1A—C3—H3C	109.5
C26—C21—N2	118.82 (15)	H3A—C3—H3C	109.5
C22—C21—N2	120.62 (14)	H3B—C3—H3C	109.5

### Hydrogen-bond geometry (Å, °)

**Table:**

Cg1 and Cg2 are the centroids of the C11–C16 and C21–C26 rings, respectively.

**Table:**

*D*—H···*A*	*D*—H	H···*A*	*D*···*A*	*D*—H···*A*
N1—H1···S1^i^	0.87 (1)	2.48 (1)	3.3240 (13)	165.(1)
N2—H2···O1	0.87 (1)	2.09 (1)	2.8993 (18)	154.(2)
C2—H2A···Cg1	0.98	3.02	3.931 (2)	155
C2—H2C···Cg2^ii^	0.98	2.80	3.607 (2)	140

Symmetry codes: (i) −*x*+2, −*y*+1, −*z*+1; (ii) −*x*+2, *y*−1/2, −*z*+3/2.
